# Desregulated microRNAs in aging-related heart failure

**DOI:** 10.3389/fgene.2014.00186

**Published:** 2014-06-25

**Authors:** Ran Zhuo, Siyi Fu, Shiyi Li, Mengchao Yao, Dongchao Lv, Tianzhao Xu, Yihua Bei

**Affiliations:** ^1^Regeneration Lab and Experimental Center of Life Sciences, School of Life Science, Shanghai UniversityShanghai, China; ^2^Shanghai Key Laboratory of Bio-Energy Crops, School of Life Science, Shanghai UniversityShanghai, China; ^3^Innovative Drug Research Center of Shanghai UniversityShanghai, China

**Keywords:** microRNAs, cardiac aging, cardiac hypertrophy, cardiac remodeling, heart failure

Heart failure is the major cause of death in the western world. Despite the development and use of standard evidence-based therapeutic strategies for heart failure like inhibition of the activity of the β-adrenergic signaling and renin-angiotensin-aldosterone system, the prevalence of heart failure is still increasing, while morbidity and mortality have not been satisfactorily improved (Hofmann and Frantz, 2013). Growing evidence has indicated that the rising incidence of heart failure is substantially associated with age. In the United States, a high proportion of the estimated 5 million heart failure patients are older people, and a vast majority of heart failure-related hospitalization and death occurred in patients over 65 years old (Go et al., 2014). With the tendency of global aging, it is necessary to go deeper in exploring the aging-related heart failure.

Cardiac aging is characterized by a series of complex events of ventricle and valvular changes involving left ventricular hypertrophy, diastolic dysfunction, increased risk of atrial fibrillation, valvular degeneration and fibrosis, and decreased maximal exercise capacity. These changes make the aged heart more susceptible to stress, leading to a high prevalence of cardiovascular diseases and heart failure (Correia et al., 2002; Dai et al., 2012a). The mechanisms of progression to heart failure in the aged heart have been previously described. The oxidative stress and mitochondrial damage are responsible for triggering the increased cardiomyocyte death including necrosis, apoptosis and autophagy, accompanied by hypertrophy of remaining cells and impaired structure of extracellular matrix (ECM), thus leading to ventricular remodeling and reduced cardiac contractility (Nadal-Ginard et al., 2003; Sarkar et al., 2004; Lindsey et al., 2006; Dai et al., 2012b; Venkataraman et al., 2013). Meanwhile, cardiac hypertrophy leads to a mismatch in oxygen supply and demand, which contributes to endothelial dysfunction and angiogenesis (Shiojima et al., 2005; Izumiya et al., 2006; Heineke et al., 2007). In response to these chronic stress, the aged heart undergoes a complex pathophysiological changes and finally progresses to symptomatic heart failure (Foo et al., 2005; Dai et al., 2012a).

MicroRNAs (miRNAs, miRs) are a novel class of small non-coding RNAs with approximately 20-24 length of base, which function as endogenous suppressors of gene expression through mRNA degradation and/or translational inhibition mainly by binding to 3′-untranslated region (3′-UTR) of target mRNAs (Lim et al., 2005; Van Rooij, 2011). Nowadays over 2000 miRNAs have been identified in human genome and each miRNA can modulate numerous target genes and build complex signaling networks (Kim and Nam, 2006; Liang, 2009). As a center player of gene regulation, many essential biological processes are regulated by miRNAs, including proliferation, apoptosis, necrosis, autophagy, differentiation, and stress responses (Bartel, 2004). Due to these multiple roles, miRNAs are critically involved in the development of multifarious heart diseases, such as heart hypertrophy, arrhythmia, acute myocardial infarction, and heart failure (Latronico and Condorelli, 2009; Xiao et al., 2012, 2014; Fu et al., 2013; Vickers et al., 2014). Several microarray studies have revealed expression profiles of specific miRNAs that are aberrantly expressed in heart failure. MiR-1, -29, -30, -133, and -150 were found to be downregulated in heart failure, whereas miR-21, -23, -27, -125, -132, -146, -195, -199, -214, -223, and 342 were upregulated (Van Rooij et al., 2006; Cheng et al., 2007; Ikeda et al., 2007; Sayed et al., 2007; Tatsuguchi et al., 2007; Thum et al., 2007; Sucharov et al., 2008; Matkovich et al., 2009; Naga Prasad et al., 2009). In addition, several circulating miRNAs including miR-423-5P have been considered as putative biomarkers for heart failure (Tijsen et al., 2010). Some distinguished reviews have summarized it in detail (Elzenaar et al., 2013; Kumarswamy and Thum, 2013; De Rosa et al., 2014; Harada et al., 2014).

As we know, cardiac aging is among the predominant risk factors for the development of heart failure (Correia et al., 2002; Dai et al., 2012a). Recent advances suggest that miRNAs may also play a role in the regulation of gene expression in cardiovascular aging processes (Zhang et al., 2012; Olivieri et al., 2013; Menghini et al., 2014). It has been previously demonstrated that 65 miRNAs were differentially expressed in the old versus young mouse adult hearts, approximately half of which belong to 11 miRNA clusters, indicating that these clusters contribute to the complex regulation of gene expression during heart aging (Zhang et al., 2012). In addition, miR-22 was shown to be involved in aging-related cardiac fibrosis, whose overexpression contributed to cellular senescence and migration of cardiac fibroblasts (Jazbutyte et al., 2013). More recently, it was demonstrated that aging-induced expression of miR-34a and inhibition of its target PNUTs lead to increased cardiomyocyte death and reduced cardiac contractility function, by inducing telomere shortening and DNA damage responses (Boon et al., 2013).

However, the role of miRNAs in aging-related heart failure is far from elucidated. A previous study showed that the members of miR-17-92 cluster, including miR-18a, -19a, and -19b, were all downregulated in failure-prone heart of aged mice as well as in cardiac biopsies of idiopathic cardiomyopathy patients at old age with severely impaired cardiac function (ejection fraction, EF<30%), accompanied by increased expression of the ECM proteins connective tissue growth factor (CTGF) and thrombospondin-1 (TSP-1). Furthermore, the *in vitro* studies showed that these expression changes were specific in aged cardiomyocytes but not in cardiac fibroblasts, and the inhibition of miR-18/19 in cardiomyocytes contributed to collagen synthesis (Collagen 1A1 and 1A3) via the regulation of pro-fibrotic CTGF and TSP-1. Although the mechanisms underlying these regulations are still unknown, it provides a close relationship between miR-18/19 and aging-induced cardiac remodeling and heart failure (Van Almen et al., 2011).

With the development of the research for roles of miRNAs in aging-related heart failure, its cellular and molecular mechanisms as well as pathophysiological changes will be further clarified, which will help develop novel miRNA-targeted therapeutic strategies for heart failure in aged people.

## Conflict of interest statement

The authors declare that the research was conducted in the absence of any commercial or financial relationships that could be construed as a potential conflict of interest.
